# Radiographic airway abnormalities in untreated early rheumatoid arthritis are associated with peripheral neutrophil activation

**DOI:** 10.1186/s13075-023-03019-5

**Published:** 2023-03-20

**Authors:** Tilia Selldén, Carina Kärrman Mårdh, Martin Joelsson, Jenny Vikgren, Åse Johnsson, Gunilla Larsson, Daniel Glinatsi, Kajsa Stubendorff, Karin Svensson, Inger Gjertsson, Anna Rudin, Anna-Carin Lundell, Anna-Karin Hultgård Ekwall

**Affiliations:** 1 Department of Rheumatology and Inflammation Research, Institute of Medicine, Sahlgrenska Academy, University of Gothenburg, Guldhedsgatan 10A, Gothenburg, SE-405 30 Sweden; 2 Department of Rheumatology, Sahlgrenska University Hospital, Gothenburg, Sweden; 3 Department of Radiology, Institute for Clinical Sciences, Sahlgrenska Academy, University of Gothenburg, Gothenburg, Sweden; 4 Department of Radiology, Sahlgrenska University Hospital, Gothenburg, Sweden; 5 Department of Rheumatology, Skaraborg Hospital, Skövde, Sweden

**Keywords:** Rheumatoid arthritis, Pulmonary abnormalities, HRCT, Neutrophil activation, Rheumatoid factor, Anti-citrullinated protein antibodies

## Abstract

**Background:**

The role of the lung for the initiation and progression of rheumatoid arthritis (RA) is still unclear. Up to 10% of RA patients develop interstitial lung disease which remains a clinical challenge. Understanding early disease mechanisms is of great importance. The objective of this study was to determine whether there is an association between peripheral neutrophil phenotypes and presence of pulmonary abnormalities (PA) on chest high-resolution computed tomography (HRCT) in untreated early RA (ueRA).

**Methods:**

Clinical data and blood were collected, and HRCT performed at diagnosis on 30 consecutive anti-citrullinated protein antibody (ACPA) and/or rheumatoid factor (RF) positive ueRA patients. HRCTs were evaluated for the presence of RA-associated parenchymal, airway and/or pleural abnormalities. Expression of phenotype markers on neutrophils were determined by flow cytometry. Levels of calprotectin, ACPA and RF were measured using immunoassays.

**Results:**

The frequency of having any PA was 60%. Airway abnormalities were present in 50%, parenchymal nodules in 43% and interstitial lung abnormalities (ILA) in 10%. Unsupervised multivariate data analysis showed clustering of any PA with neutrophil activation, parameters of inflammation and RF titres. In univariate analysis, the patients with PA displayed significantly increased CD11b and decreased CD62L expression on neutrophils (1.2-fold, *p* = 0.014; 0.8-fold, *p* = 0.012) indicating activation and significantly increased RF IgM titre and CRP (5.7-fold, *p* = 0.0025; 2.3-fold, *p* = 0.0035) as compared to no PA. Titres of RF, but not ACPA, correlated with expression of the neutrophil activation marker CD11b. A stratified analysis demonstrated that airway involvement was the PA subtype with the strongest association with neutrophil activation.

**Conclusion:**

We report a strong association between radiographic airway findings and activation of circulating neutrophils in early RA supporting a role of innate immunity and the lung at disease onset. Our results also indicate different contributions of RF and ACPA in the RA pathogenesis.

**Supplementary Information:**

The online version contains supplementary material available at 10.1186/s13075-023-03019-5.

## Background

Rheumatoid arthritis (RA) is a systemic inflammatory autoimmune disease primarily affecting joints [1]. However, approximately 60% of the patients will develop pulmonary involvement [2]. Some manifestations are asymptomatic, whereas other progress to a life-threatening disease [3]. High-resolution computed tomography (HRCT)-detected pulmonary abnormalities (PA) of parenchyma and airways are more prevalent in recent onset RA than in healthy controls (age and sex matched) independently of smoking [4]. RA is believed to originate at mucosal sites such as the airways, and several lines of evidence suggest that the break of tolerance and initiation of autoimmunity takes place in the lung as a consequence of repeated environmental stress in individuals with a susceptible genetic background [5]. Reported risk factors for pulmonary disease in RA include smoking, presence of autoantibodies and disease duration [2]. It is not understood why some RA patients develop pulmonary manifestations and others not and why some patients suffer from airway disease and others have isolated interstitial involvement.

Approximately 10% of RA patients will develop clinically significant RA-associated interstitial lung disease (RA-ILD) over the course of the disease, but sub-clinical interstitial lung abnormalities (ILA) have been described in up to 44% of the patients [6, 7]. Furthermore, pulmonary nodules and signs of airway disease (bronchiolitis and/or bronchiectasis) are more prevalent in RA compared to healthy controls [4, 8]. Obliterative bronchiolitis and ILD are severe extra-articular manifestations of RA with high morbidity and mortality [2]. Therapy for these progressive conditions is limited and can at best reduce the rate of decline in lung function. Together, this emphasizes the need for increased knowledge on pulmonary disease mechanisms as well as biomarkers for early and subtype-specific lung diagnoses.

Neutrophils have emerged as a key player in RA based on their capacity to drive inflammation, induce and promote adaptive immune responses and mediate tissue damage [9]. Circulating neutrophils in RA display an activated phenotype with increased production of neutrophil extracellular traps (NETs) [10]. Anti-citrullinated protein antibodies (ACPAs) and antibodies to immunoglobulins (rheumatic factor; RF) are significant predictors of ILD-development in RA [2, 11]. These autoantibodies form immune complexes which are strong inducers of inflammatory responses of neutrophils [9]. In a recent study across different RA populations, neutrophil-derived calprotectin was superior to C-reactive protein (CRP) in identifying patients with active disease, and a neutrophil biomarker panel demonstrated good predictive value for extra-articular nodules [12].

Expression of the surface neutrophil glycoprotein CD177 reportedly promote development of some autoimmune diseases but is protective for others [13]. Low-density neutrophils have been suggested to represent an immature neutrophil population in RA with enhanced survival and defective TNF signalling possibly leading to lower response to therapy [14]. The role of different neutrophil phenotypes in the development of pulmonary disease in RA has not been clarified.

The objective of the present study was to determine if there is an association between neutrophil phenotypes in peripheral blood and presence of pulmonary abnormalities (PA) on chest high-resolution computed tomography (HRCT) in untreated early RA (ueRA).

## Patients and methods

### Study population and design

Thirty consecutive adult patients with recent onset seropositive and disease-modifying anti-rheumatic drug (DMARD)-naïve RA fulfilling the 2010 ACR/EULAR RA classification criteria were included at the time of diagnosis in this cross-sectional study. Seropositivity was defined as a presence of RF (IgA and/or IgM) and/or ACPA. Study activities included medical history, demographics, concomitant medication, smoking habits, patient and physician global assessments of disease activity, swollen and tender joint counts, pulmonary HRCT, radiography of hands- and feet and blood sampling. All study activities were performed within a week, in most cases within 72 h, from diagnosis before initiation of disease-modifying antirheumatic drugs (DMARDs) including oral prednisolone. Patients with a history of oral glucocorticoid use were eligible if treatment had been discontinued ≥ 2 weeks before inclusion. The use of NSAID was allowed. Symptom duration was defined as time in months since self-reported onset of first disease-associated symptoms. Patients with symptom duration > 36 months were excluded. Additional exclusion criteria included any contraindication for HRCT, current active joint disease other than RA, history of malignancy within 5 years, immuno-deficiency, active infection requiring anti-infectives within 2 weeks of inclusion and highly active RA in need of urgent treatment.

### Patient data and clinical assessments

Demographic data (age, sex and smoking status) and self-reported history of pulmonary disease (such as chronic bronchitis, asthma, chronic obstructive pulmonary disease and lung cancer) were collected, as well as clinical parameters of disease activity (number of swollen and tender joints of 28 joints, patient global assessment (PGA) of disease activity (VAS 0–100 mm), physician global assessment (PhGA) of disease activity (VAS 0–100 mm), CRP, ESR, presence of joint erosions on radiographs of hands and feet) and levels of autoantibodies (RF IgM, RF IgA and ACPA) (Table 1). Smoking status was defined as follows: *current smoker* if ever smoked ≥ 100 cigarettes AND is currently smoking, *former smoker* if ever smoked ≥ 100 cigarettes AND no smoking for the last 12 months, *never smoker* if smoked less than 100 cigarettes during a lifetime.

**Table 1:** Patient demographics and clinical characteristics

Variables	Untreated early RA patients (*n* = 30)
Age, median (range), years	58 (22–75)
Female sex, *n* (%)	25 (83%)
Smoking status, *n* (%)	
Never smoker	18 (60%)
Former smoker	6 (20%)
Current smoker	6 (20%)
Self-reported lung disease history, *n* (%)	4 (13%)
Symptom duration, median (range), months	6 (1–31)
Disease activity, *n* (%)	
SJC28, median (range)	7 (1–17)
TJC28, median (range)	7 (0–16)
ESR (mm), median (range)	19.5 (2–104)
CRP (mg/L), median (range)	7.6 (0.7–105)
DAS28, mean (± SD)	4.9 (± 1.1)
DAS28-CRP, mean (± SD)	4.6 (± 1.0)
CDAI, mean (± SD)	23.6 (± 9.1)
SDAI, mean (± SD)	25.4 (± 10.1)
Joint erosion, *n* (%)	7 (24%)
RF positive, *n* (%)^a^	22 (73%)
ACPA positive, *n* (%)^b^	23 (77%)
RF and ACPA positive, *n* (%)^a,b^	16 (53%)

*RA* Rheumatoid arthritis, *SJC28* Swollen joint count in 28 joints, *TJC28* Tender joint count in 28 joints, *CRP* C-reactive protein, *ESR* Erythrocyte sedimentation rate, *DAS28* Disease activity score in 28 joints with ESR, *CDAI* Clinical disease activity index, *SDAI* Simple disease activity index, *RF* Rheumatoid factor, *ACPA* Anti-citrullinated protein antibodies. Symptom duration is defined as time since appearance of patient reported RA symptoms

^a^RF IgM levels > 5 kIE/L and/or RF IgA > 20 kIE/L are considered positive

^b^ACPA level > 5 U/mL is considered positive

### High-resolution computed tomography (HRCT)

HRCT was performed using a Siemens SOMATOM Force scanner (Siemens Healthineers, Germany) within 1 week before any treatment was initiated. Volumetric CT scans were achieved in maximal inspiration and expiration using a low dose protocol: 120 kV, reference mAs 30, acquisition 192 × 0.6 mm with a pitch of 0.9, rotation time 0.5 s applying 4D-Care dose and ADMIRE (strength 3). Images with a slice thickness of 0.75 mm were reconstructed with kernels Bf32 and BI64. No contrast was given. All images were evaluated for abnormalities by two experienced thoracic radiologists in consensus. RA-associated HRCT findings were categorized as ILA by the presence of reticulations, ground-glass opacities, honeycombing and/or consolidations; pulmonary nodules by the presence of parenchymal nodule(s) ≥ 3 mm; airway abnormalities by the presence of air trapping and/or bronchial wall thickening (bronchiolitis) and/or bronchiectasis; and pleural abnormalities by the presence of pleural thickening and/or effusion. Pulmonary nodules were quantitatively evaluated by means of computed aided detection and semiautomatic nodule segmentation using Syngo. CT Lung CAD, software version VC20G release VC 20 (Siemens Healthcare GmbH, Germany). All patients with parenchymal nodules ≥ 6 mm were subjected to follow up according to national guidelines for lung cancer. No patient was diagnosed with cancer based on the initial HRCT performed as part of this study. No evaluation of the vasculature was performed.

### Neutrophil characterization

Blood samples were collected in heparin tubes immediately before HRCT (i.e. within 1 week from diagnosis and before initiation of treatment). All analyses were performed within 6 h. Neutrophils were separated using density centrifugation (Ficoll-Paque Plus, Cytiva, Sweden), in this paper referred to as low-density granulocytes (LDG) and normal density granulocytes (NDG), and subjected to flow cytometry as previously described [15]. Activation of neutrophils was assessed by expression of CD11b and CD62L. 7-aminoactinomycin D (7-AAD) was used to separate dead from live cells. The proportion of LDGs in the peripheral blood mononuclear cell layer was determined based on CD45 expression and side scatter characteristics. The mean fluorescence intensity (MFI) of the surface markers CD62L, CD11b and CD177 were determined. Gating strategy is presented in Additional file 1, figure S1. All samples were acquired in a FACSVerse equipped with FACSuite Software (BD Biosciences, Franklin Lakes, USA) and analysed with the FlowJo Software (TreeStar, Ashland, Oregon, USA). All antibodies used for neutrophil characterization are listed in Additional file 2, table S1.

### Autoantibodies and markers of inflammation

Levels of RF were measured using EliA test on Phadia 250 (Thermo Fisher, Waltham, USA). Anti-CCP IgG (ACPA) levels were measured using chemiluminescent microparticle immunoassay on the Alinity i-platform (Abbott Laboratories, Chicago, USA). CRP was analysed using Turbidimetry Alinity and ESR was analysed according to standard lab protocols. Serum calprotectin (S100A8/A9) levels were analysed using particle-enhanced turbidimetric immunoassay (Gentian Diagnostics, Moss, Norway) on an Optilite system (Binding Site, Birmingham, England).

### Statistical analysis

Multivariate data analysis was performed using the SIMCA-P software version 17.0.2 (Sartorius Stedim Biotech, Goettingen, Germany). Principal component analysis (PCA) was used to make an unsupervised dimensionality reduction analysis to explore and visualise the overall relationship between neutrophil activation status, neutrophil subtypes, calprotectin, disease activity measures and demographic data. Only binary data (presence or not) of radiographic HRCT findings were included in the analysis. DAS28, DAS28-CRP, CDAI and SDAI were not included since these disease activity measures are calculated form other variables included. Orthogonal partial least squares discriminant analysis (OPLS-DA) was implemented to find the most influential variables driving the distinction between two pre-defined groups based on presence or subtype of pulmonary abnormalities. Default settings for the PCA-X and OPLS-DA models were used; data were centred and scalded to unit-variance (UV) in the software to give all variables equal weight. Variable influence on projection (VIP) values were used to discriminate between relevant and less relevant X-variables for each respective OPLS-DA model (Additional file 1, figure S2). The column loading plots presented in Figs. 2A and 5A consist of variables with VIP-values ≥ 0.95 or VIP-values ≥ 1.0 respectively. Model quality was based on R2 and Q2 parameters and are presented in each figure.

The most relevant X-variables, as indicated by larger bars and smaller error bars in the OPLS-DA column loading plots, were further analysed using univariate statistical analysis. Non-parametric Mann–Whitney *U* test and Spearman’s correlation test were used for sample sets of continuous variables and Fisher’s exact test was used for categorical variables (GraphPad Prism software version 9.4.0, San Diego, California USA). *p*-values equal or less than 0.05 were considered significant and *, **, *** indicate *p* ≤ 0.05, *p* ≤ 0.01 and *p* ≤ 0.001 respectively.

## Results

### Airway abnormalities are the most prevalent HRCT findings in ueRA

Demographic and clinical data are summarized in Table 1. The median age of the RA patients was 58 years, 83% were female, and the median patient-reported symptom duration was 6 months. Four patients reported history of lung disease (all cases being asthma). Among the RA patients, 60% were never smokers and 20% were currently smoking. The mean disease activity was moderate by DAS28 (4.9) but high by CDAI (23.6) composite scores. Seventy-three percent of the patients were positive for RF (IgM or IgA), 77% were positive for ACPA, and 53% of the patients were double-positive.

The frequency of having any RA-associated PA by HRCT was 60% (18 out of 30 patients) (Table 2). Airway abnormality was the most prevalent HRCT finding in this ueRA cohort (53%). Two patients had bronchiectasis and 14 patients (47%) showed signs of bronchiolitis (bronchial wall thickening and/or air trapping). Parenchymal nodules were found in 13 patients (43%). The mean number of nodules was 1 (range 0–9), and the mean total nodule volume per patient was 48 (range 26–480) mm^3^. Radiological signs of ILA were uncommon (10%), and all these patients had mild findings of reticulations. No patients showed radiological signs of pleural involvement. Representative images of the findings are included in Additional file 1, figure S3.

**Table 2:** RA-associated pulmonary abnormalities by HRCT at diagnosis

RA-associated pulmonary abnormalities (PA)	
Any RA-associated PA, *n* (%)	18 (60%)
Airway abnormalities, *n* (%)	15 (50%)
Bronchiectasis	2 (7%)
Bronchiolitis	14 (47%)
Bronchial wall thickening	4 (13%)
Air trapping	13 (43%)
Parenchymal nodule, *n* (%)^a^	13 (43%)
Interstitial lung abnormalities, *n* (%)	3 (10%)
Reticulations	3 (10%)
Ground glass opacities	0
Honeycombing	0
Consolidations	0
Pleural disease, *n* (%)	0
Effusion	0
Pleural thickening	0

^a^Parenchymal nodule was defined as ≥ 3 mm

### Pulmonary abnormalities are associated with a peripheral activated neutrophil phenotype

**Fig. 1:**
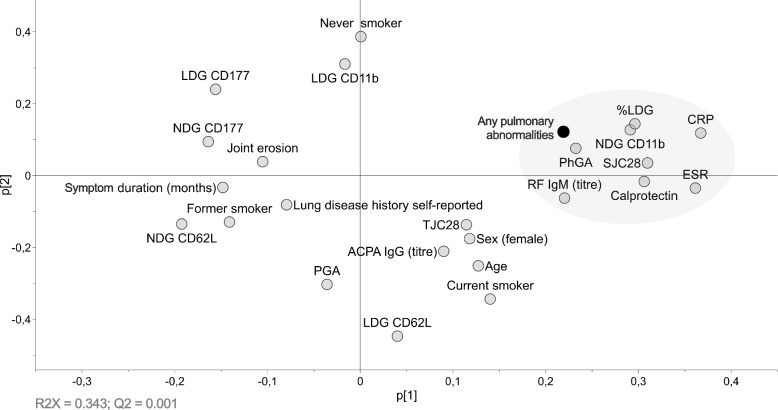
Principal component analysis (PCA) plot showing an overview of the relationship between the presence of PA by HRCT (Any pulmonary abnormalities), neutrophil activation status (CD11b and CD62L expression) and subtypes (% LDG and CD177 expression) on LDGs and NDGs, calprotectin, as well as disease activity measures and demographic data in ueRA patients(*n* = 30). Closely clustered variables to Any pulmonary abnormalities (black circle) indicate positive association (grey area), while diagonally opposite variables indicate inverse relationship. Flow cytometry variables are represented as MFI

It is unknown whether peripheral neutrophil phenotypes are related to pulmonary pathology in patients with ueRA. First, unsupervised PCA was used to make unbiased cluster analysis of collected data including; presence of any PA by HRCT, neutrophil activation status (CD11b and CD62L expression), neutrophil subtypes (% LDG and CD177 expression) and neutrophil-derived proteolytic enzyme (calprotectin), as well as disease activity measures and demographic data (Fig. 1). *Any PA* was clustered together with high expression of CD11b (indicating activation) on NDGs, high frequency of LDGs and calprotectin indicating positive association between these variables. This cluster also included RF IgM levels and the clinical parameters of inflammation (CRP, ESR, number of swollen joints (SJC)) and the PhGA. Importantly, history of lung disease or symptom duration did not associate with the PA cluster suggesting that the PA of this study population and neutrophil activation are early events in RA. ACPA levels and current smoker were not clustered with *Any PA* in this analysis.

**Fig. 2:**
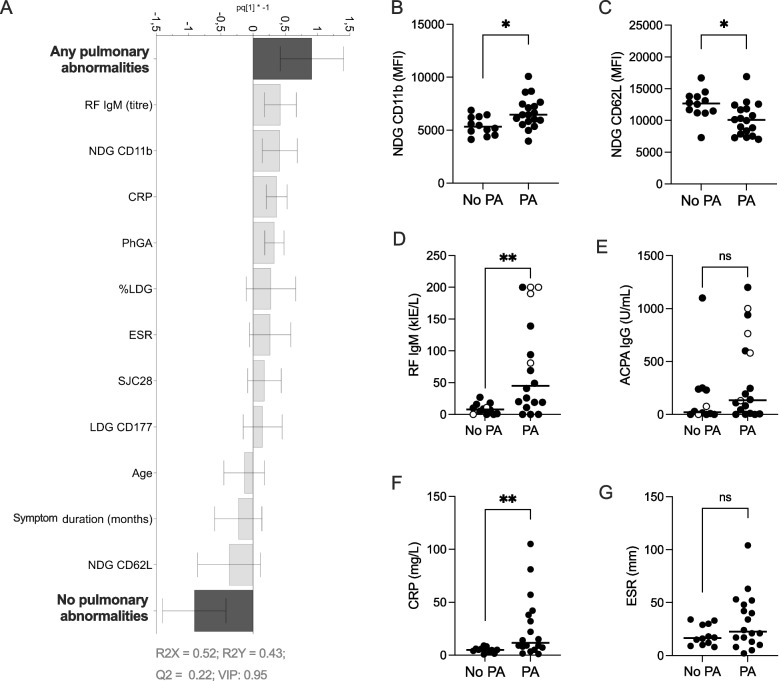
OPLS-DA column loading plot (VIP > 0.95) demonstrating the difference between the two patient groups *Any pulmonary abnormalities (Any PA)* and *No pulmonary abnormalities (No PA)* with respect to neutrophil activation status (CD11b and CD62L expression) and subtypes (% LDG and CD177 expression) on LDGs and NDGs, calprotectin, as well as disease activity measures and demographic data (**A**). Univariate analysis of NDG CD11b (**B**), NDG CD62L (**C**), RF (**D**), ACPA (**E**), CRP (**F**) or ESR (**G**) in patients with *Any PA* compared to *No PA*. Open circles in **E**–**F** indicate current smokers. Bars show median. ns, non-significant, *p ≤ 0.05, **p ≤ 0.01 (Mann–Whitney *U* test)

Next, an OPLS-DA analysis was performed to identify the variables discriminating the group *Any PA* from the group *No PA*. *Any PA* demonstrated a positive association with RF IgM, high expression of CD11b on NDGs, and CRP, and negative association with high expression of CD62L on NDGs (Fig. 2A). Subsequent univariate analysis confirmed that RA patients with *Any PA* had significantly increased CD11b expression on NDGs (MFI 6455 vs 5317, *p* = 0.014) and significantly lower expression of CD62L on NDGs (MFI 10,065 vs 12,676, *p* = 0.012), indicating neutrophil activation, as compared to *No PA* (Fig. 2B, C). The LDG proportions were higher in *Any PA* compared to *No PA*, but the proportions were low in both groups (< 4%) except for one outlier in *Any PA* (Additional file 1, figure S4A).

### Pulmonary abnormalities are associated with levels of RF but not levels of ACPA

A significant increase in levels of RF (75.4 vs 8.6, *p* = 0.0025), but not ACPA (336.1 vs 162.5 *p* = 0.17), was found in patients with *Any PA* as compared to *No PA* (Fig. 2D, E). When excluding current smokers (open circles in Fig. 2D, E) in the analyses, RF levels were still higher but not ACPA (*p* = 0.034 and *p* = 0.62), which indicates that smoking status could not explain the differences in RF levels. In addition, patients with *Any PA* had higher levels of CRP (11.5 vs 5.0, *p* = 0.0035) compared to *No PA* patients, while there was no significant difference in ESR between groups (Fig. 2F, G). The *Any PA* and *No PA* groups did not differ in disease activity measures CDAI, SDAI, SCJ28, TCJ28 and DAS28, nor with respect to smoking status: never, former or current (Additional file 2, table S2). Furthermore, there were no significant differences in calprotectin serum levels in patients with versus without PA nor in patients with versus without joint erosions (Additional file 1, figure S4 B-C). Thus, presence of PA in this study population was associated with CRP, high RF IgM titres and neutrophil activation.

**Fig. 3:**
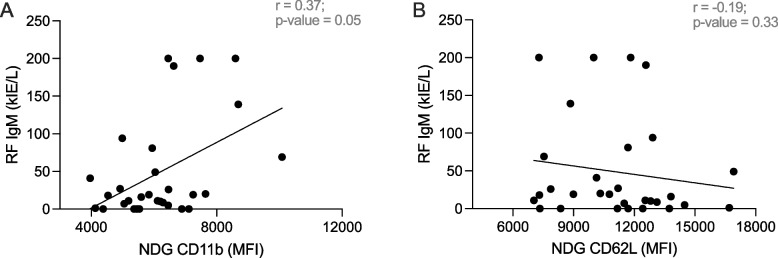
Correlation analysis between RF IgM titres and neutrophil activation status; NDG CD11b (**A**) and NDG CD62L (**B**) expression in RA patients (*n* = 30). ns, non-significant, *p ≤ 0.05 (Spearman’s rank correlation test). A linear regression line is presented in the respective plot

The fact that both RF IgM titres and markers of neutrophil activation in this ueRA cohort were associated with the presence of PA prompted us to investigate if there is a correlation between these two factors. Indeed, there was a significant positive correlation of RF IgM titres and CD11b expression on NDGs (*r* = 0.37, *p* = 0.05) (Fig. 3A). However, no significant correlation was found using loss of CD62L as a marker of activation (*r* =  − 0.19, *p* = 0.33) (Fig. 3B). No correlation was found between levels of ACPA IgG and mean fluorescence intensity of CD11b nor CD62L on NDGs (Additional file 1, figure S5).

### Airway abnormality is the main subtype behind the association of PA, neutrophil activation and RF titres

**Fig. 4:**
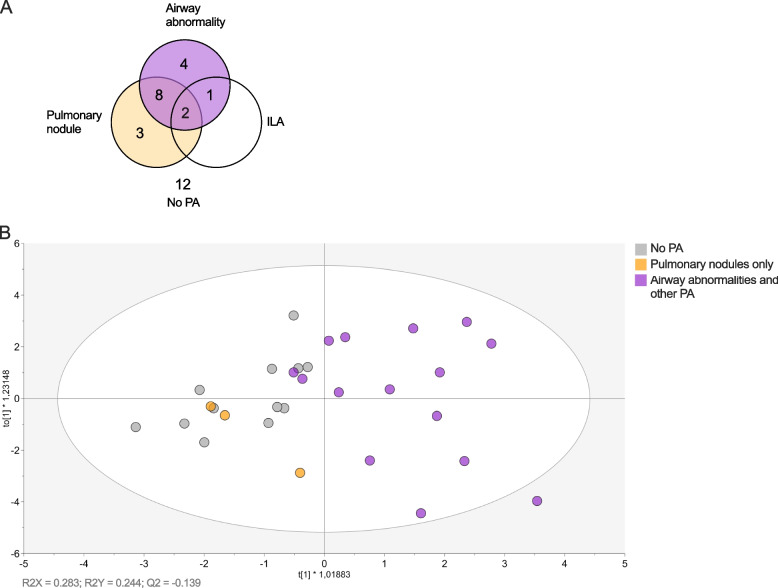
Venn diagram showing the overlap of the subtypes of PA (**A**). OPLS-DA score plot showing the separation of patients belonging to each PA subtype group; Airway abnormalities (including patients with coexisting nodules and/or ILA), Pulmonary nodules only (excluding patients with coexisting airway abnormalities) and No PA in the RA patients (n=30) (**B**). Closely clustered patients indicate similarities in neutrophil activation status (CD11b and CD62L expression), neutrophil subtypes (% LDG and CD177 expression), calprotectin, as well as disease activity measures and demographic data

The overlap between the radiographic entities is illustrated by a Venn diagram in Fig. 4A. The vast majority (15/18) of patients with PA had airway abnormalities. Three patients with pulmonary nodules did not have any other PA. All patients with ILA also had airway abnormalities by HRCT. The small size of the ILA group precluded any further analysis with respect to this radiographic finding in this study.

To further elucidate which specific radiographic finding is the major driver for the association of *Any PA* with neutrophil activation, we performed an OPLS-DA analysis in which the three non-overlapping sub-groups; *Airway abnormalities* (including patients with coexisting nodules and/or ILA), *Pulmonary nodules only* (excluding patients with coexisting airway abnormalities) and *No PA*. The OPLS-DA score-plot demonstrated a separation between patients with *Airway abnormalities* and patients with *No PA*, indicating that these groups are different form each other with respect to neutrophil activation status, neutrophil subtypes, calprotectin, disease activity and demographic data. On the other hand, patients with *Pulmonary nodules only* clustered together with *No PA* indicating similarities between the groups (Fig. 4B).

**Fig. 5:**
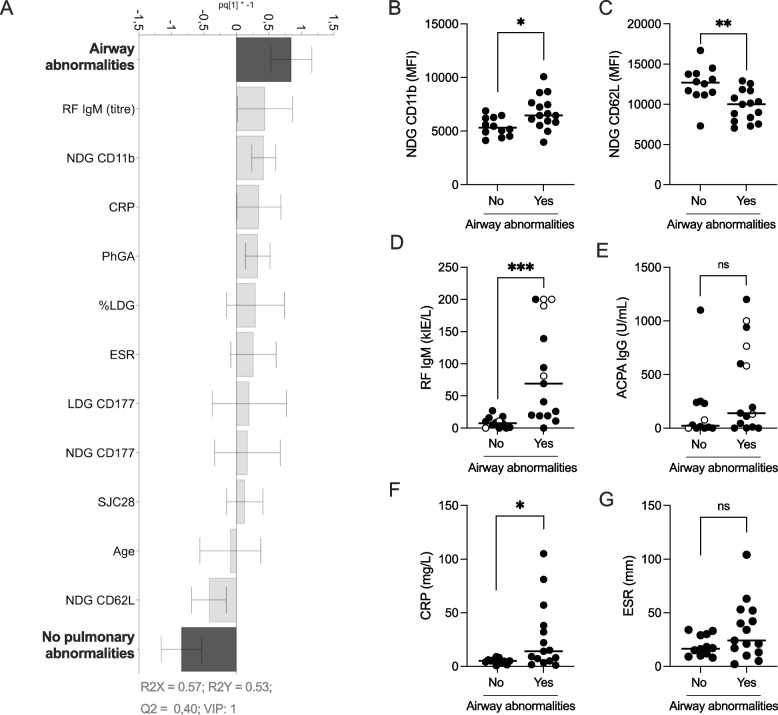
OPLS-DA column loading plot (VIP > 1.0) demonstrating the difference between the two patient groups *Airway abnormalities* (including patients with coexisting nodules and/or ILA) and *No PA*, with respect to neutrophil activation status (CD11b and CD62L expression), neutrophil subtypes (% LDG and CD177 expression), calprotectin, as well as disease activity measures and demographic data (**A**). Subsequent univariate analysis of airway abnormalities and CD11b (**B**), CD62L (**C**), RF (**D**), ACPA (**E**), CRP (**F**) and ESR (**G**). Bars show median. ns, non-significant, *p ≤ 0.05, **p ≤ 0.01, *** *p* ≤ 0.001 (Mann–Whitney *U* test)

Discriminant analysis of the two groups *Airway abnormalities* and *No PA* using OPLS revealed that *Airway abnormalities* was associated with the expression of CD11b on NDGs and RF IgM, and *No PA* with the expression of CD62L on NDGs, i.e. less neutrophil activation (Fig. 5A) (good quality (R2X > 0.4) and *goodness of fit* (R2Y > 0.5) of the model). Subsequent univariate analysis demonstrated a significant increase in CD11b (1.3-fold, *p* = 0.014) (Fig. 5B) and decrease in CD62L (0.6-fold, *p* = 0.003) (Fig. 5C) expression on NDGs in patients with airway abnormalities as compared to no pulmonary abnormalities. Furthermore, the group *Airway abnormalities* had significantly higher levels of RF IgM (8.8-fold, *p* = 0.0002) (Fig. 5D), but not ACPA, titres (*p* = 0.16) (Fig. 5E) compared to *No PA*. Again, excluding current smokers (open circles in Fig. 5D, E) from the analyses indicated that smoking status could not explain the differences in RF titres (RF *p* = 0.004 and ACPA *p* = 0.65). As in the non-stratified analysis, CRP levels were significantly higher (2.8-fold, *p* = 0.013), but not ESR (*p* = 0.14) (Fig. 5F, G), in *Airway abnormalities* as compared to *No PA*. Taken together, the stratified analysis indicates that airway abnormality is the major subtype behind the association of PA with high levels of RF IgM and neutrophil activation.

## Discussion

Lung disease remains one of the most therapeutically challenging extraarticular manifestations in RA and cause increased morbidity and premature mortality [16]. This is the first study that investigates, and provides evidence for, an association of peripheral neutrophil activation and pulmonary abnormalities, in particular signs of airway involvement, in ueRA. Our data supports a role for innate immunity and the lung in RA disease pathogenesis and confirms earlier reported fact that circulating neutrophils are more activated in RA [12]. Furthermore, our data highlights the importance of RF in disease pathogenesis, and it is tempting to speculate that RFs might participate in priming of neutrophils.

Neutrophils are key modulators of innate and adaptive immune responses during acute inflammation and infections, as well as of the resolution of inflammation of affected tissues [9]. In the joints in RA, neutrophils accumulate and become activated by immune complexes such as RFs and evidence suggest degranulation and NETosis by inflammatory neutrophils lead to activation of stromal and immune cells, ACPA formation and cartilage destruction [10]. Interestingly, all patients in the present study had active arthritis but only the individuals with arthritis and PA had sign of peripheral neutrophil activation.

Increasing evidence support that inflammatory events and immune activation, including NETosis and ACPA formation, at mucosal sites such as in the airways are key steps in the initiation of RA [4, 5, 17–20]. Our findings that radiographic signs of airway inflammation are associated with neutrophil activation and systemic inflammation in untreated RA patients at time of diagnosis support this hypothesis. Whether this initial inflammatory activity, predominantly of airways, in early RA will resolve by treatment, or progress further to lung disease of different types is not answered by the present data.

The significant correlation of RF titres and the expression of a neutrophil activation marker is interesting. Neutrophils have been reported to be activated by both RF and ACPAs in vitro [21, 22]. Our data, suggest that RF might be more important for neutrophil activation and for airway inflammation than ACPAs in early RA. The latter agrees with findings from the Swedish LURA study, where the frequencies of airway abnormalities were equal in ACPA + and ACPA − patients [11]. Furthermore, in agreement with earlier reports, the PCA of our cohort data did not cluster current smokers with Airway abnormalities [11] but support close association between smoking, arthralgia (tender joint count) and ACPAs [23].

The increased activation of neutrophils in patients with PA was found in normal density granulocytes but not in LDGs. The proportion of LDGs in this ueRA cohort was low compared to systemic lupus erythematosus [15]. Whether there is an association of frequency and/or activation of LDGs and lung disease in RA needs to be evaluated in further studies. According to Bach et al., neutrophil-derived calprotectin was superior to CRP in identifying patients with active disease [12]. However, the levels of calprotectin were not different in patients with PA as compared to no PA in our study and we could not confirm the reported association of calprotectin and joint erosion which was reported for established RA [12].

The frequency of PA found in association with RA varies greatly between studies and most studies have been performed on established RA with years of treatment at inclusion and the potential impact of different DMARDs on lung disease development has still not been clarified. A key strength of our study is that HRCT assessments and neutrophil analyses were collected on patients at the time of RA diagnosis, using the same HRCT equipment and laboratory facility, and before initiation of any DMARD or glucocorticoid therapy. Neutrophil analysis was performed fresh on the same day (same time point for all samples) limiting influence of auto-activation in vitro. In the present cohort, most of the patients had RA-associated PA on HRCT, and the prevalence of the different subtypes are in overall agreement with the Swedish LURA study [4]. Another strength of our study is that an unsupervised approach was used to evaluate the multidimensional patient data. This strategy resulted in an improved separation of the different radiographic entities. A limitation of our study is the relatively small sample size, which resulted in low numbers of patients with ILA and limited the possibilities for further analysis on this group. Furthermore, the frequency of females was higher in our cohort compared to other Nordic ueRA cohorts and the findings must be evaluated accordingly. Finally, the lack of clinical samples and neutrophil data from lung tissue prevented further correlation analysis of neutrophil phenotypes of the blood with that of airways.

## Conclusions

We report an association between the presence of PA on HRCT and increased activation status of circulating neutrophils at diagnosis in patients with ueRA. Furthermore, our data demonstrate a significant correlation between higher levels of RF, but not levels of ACPA, and airway abnormalities suggesting that these autoantibodies mediate different disease-associated processes. Taken together, our data supports a role for innate immunity and the lung in RA disease pathogenesis. Further studies are needed to elucidate the role of neutrophils in the development of RA-associated progressive lung disease.

## Supplementary Information

**Additional file 1: Supplementary figure 1. **Gating strategy of flowcytometry data analysis, **Supplementary figure 2**. VIP data, **Supplementary figure 3**. A High-Resolution Computed Tomography (HRCT) scan, **Supplementary figure 4**. A) Univariate analysis of % LDG in Any PA vs No PA, B) calprotectin levels in Any PA vs No PA and C) calprotectin levels in joint erosions vs No joint erosions, **Supplementary figure 5**. Correlation analysis of ACPA levels and neutrophil activation markers CD11b (A) and CD62L (B).

**Additional file 2: Supplementary table 1.** Cluster of differentiation markers used for neutrophil characterization. **Supplementary table 2.** Demographics and clinical data in patients with versus without RA-Associated PA.

**Additional file 3. **Source dataset.

## Data Availability

The datasets supporting the conclusions of this article are included within the article and additional files.

## Abbreviations

ACPA: Anti-citrullinated protein antibodies
CDAI: Clinical disease activity index
CRP: C-reactive protein
DAS28: Disease activity score, 28 joints
DMARD: Disease modifying anti-rheumatic drug
ESR: Erythrocyte sedimentation rate
HRCT: High-resolution computed tomography
EULAR: European league against rheumatism
ILA: Interstitial lung abnormality
LDG: Low density granulocyte
NGD: Normal density granulocyte
NET: Neutrophil extracellular traps
OPLS-DA: Orthogonal partial least squares discriminant analysis
PA: Pulmonary abnormality
PBMC: Peripheral blood mononuclear cell
PCA: Principal component analysis
PGA: Patient global assessment of disease activity
PhGA: Physicians’ global assessment of disease activity
RA: Rheumatoid arthritis
RF: Rheumatoid factor
SJC-28: Swollen joint count of 28 joints
SDAI: Simple disease activity index
TJC-28: Tender joint count of 28 joints
ueRA: Untreated early rheumatoid arthritis
VIP: Variable influence on projection
