# Supplementary material for: Radiographic airway abnormalities in untreated early rheumatoid arthritis are associated with peripheral neutrophil activation
Source: Arthritis Res Ther. 2023 Mar 20;25:44. doi: 10.1186/s13075-023-03019-5 (PMC10026468; doi:10.1186/s13075-023-03019-5)
Supplement: Supplementary file 1 — Additional file 1: Supplementary figure 1. Gating strategy of flowcytometry data analysis, Supplementary figure 2. VIP data, Supplementary figure 3. A High-Resolution Computed Tomography (HRCT) scan, Supplementary figure 4. A) Univariate analysis of % LDG in Any PA vs No PA, B) calprotectin levels in Any PA vs No PA and C) calprotectin levels in joint erosions vs No joint erosions, Supplementary figure 5. Correlation analysis of ACPA levels and neutrophil activation markers CD11b (A) and CD62L (B). [file 13075_2023_3019_MOESM1_ESM.pdf]

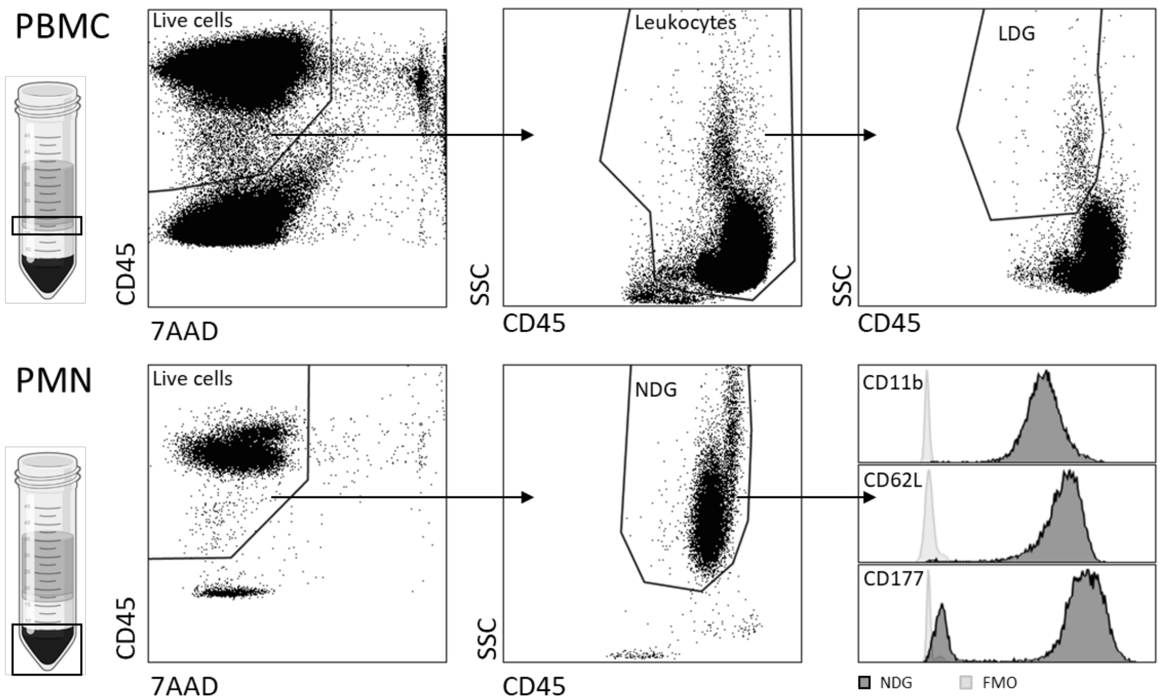

Supplementary Figure S1. Gating strategy for flow cytometry analysis of the percentage of low-density granulocytes (LDG) among peripheral blood mononuclear cells (PBMC), and of markers in normal-density granulocytes (NDG). A similar analysis of markers was performed for LDG. After density centrifugation of whole blood, LDG are found in the PBMC fraction and NDG are found in the fraction of polymorphonuclear cells (PMN). Dead cells were excluded by 7AAD staining. In the PBMC sample, all leukocytes were gated to see the percentage of LDG among leukocytes in PBMC. The granulocyte populations in PMN and PBMC were selected by CD45 and side scatter. Doublets were removed using FSC-H and FSC-A. NDG and LDG expressions of CD11b, CD62L and CD177 were measured by mean fluorescence intensity (MFI).

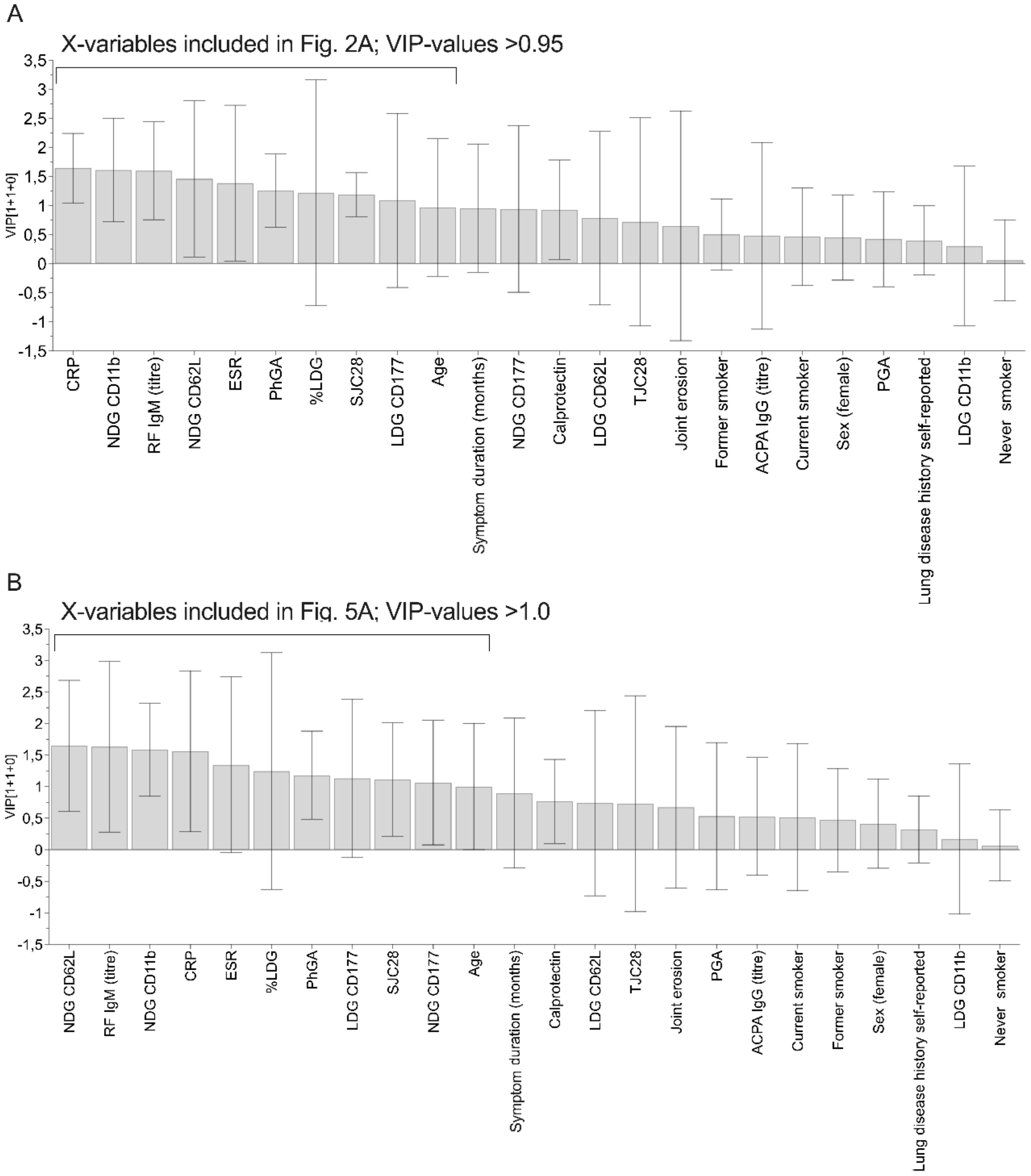

Supplementary Figure S2. Variable influence on projection (VIP) in the OPLS-DA analysis. VIP is a cumulative measure of the influence of each X-variable on the OPLS-DA model. This figure shows the VIP models for the OPLS-DA analysis with Any PA vs No PA **(A)**, Airway abnormalities vs No PA as Y-variable **(B)**. X-variables (bars in grey) in all OPLS-DA analysis include neutrophil activation status (CD11b and CD62L), neutrophil subtypes (LDG and CD177 expressing cells), calprotectin, disease activity measures and demographic data. The VIP limit for exclusion was chosen to >0.95 and >1.0 as indicated.

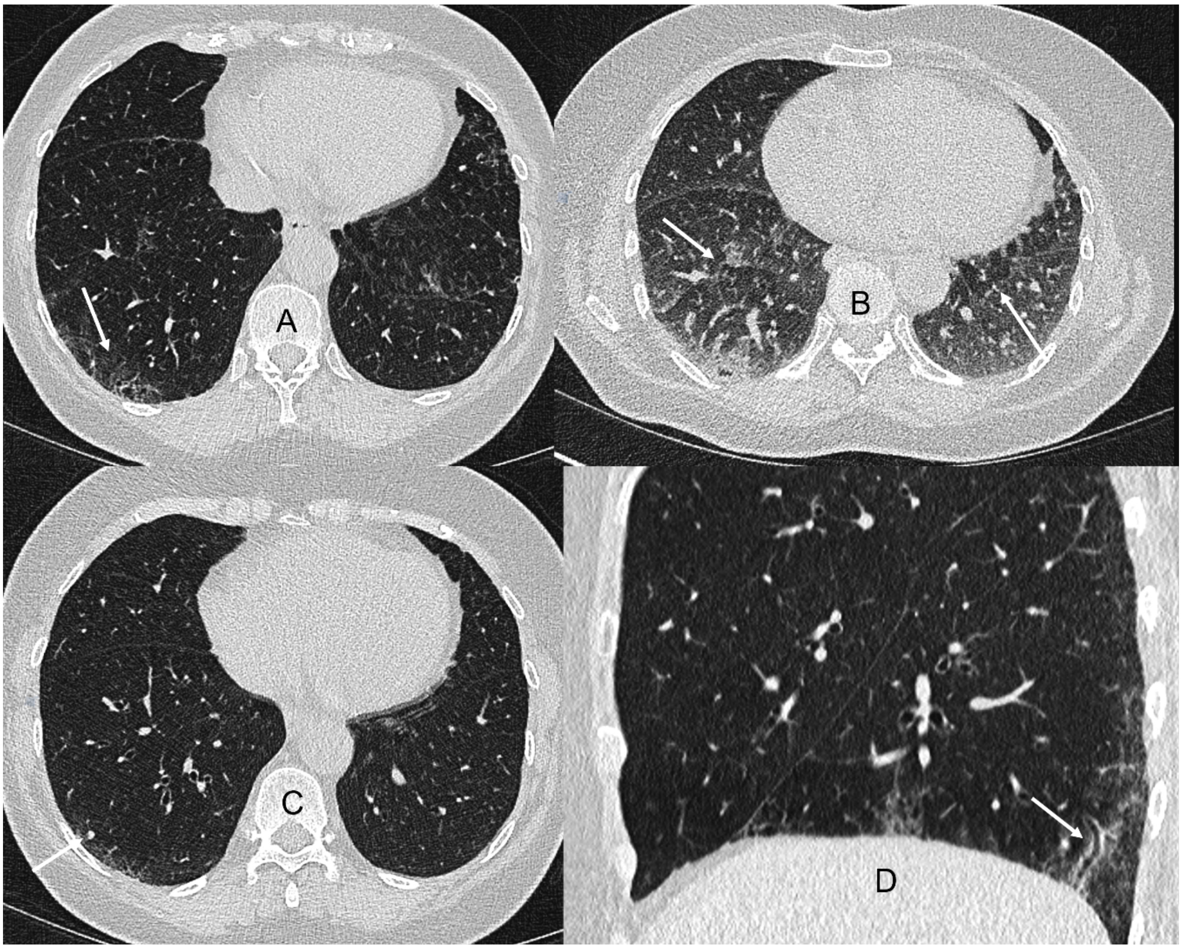

Supplementary Figure S3. A High-Resolution Computed Tomography (HRCT) scan of one of the study participants illustrating reticulation in the inspiratory scan **(A),** air trapping in the expiratory scan **(B)** marked with arrows, a small nodule marked with an arrow **(C)** and a dilated bronchus with increased wall thickness marked with an arrow in a sagittal reformat **(D).**

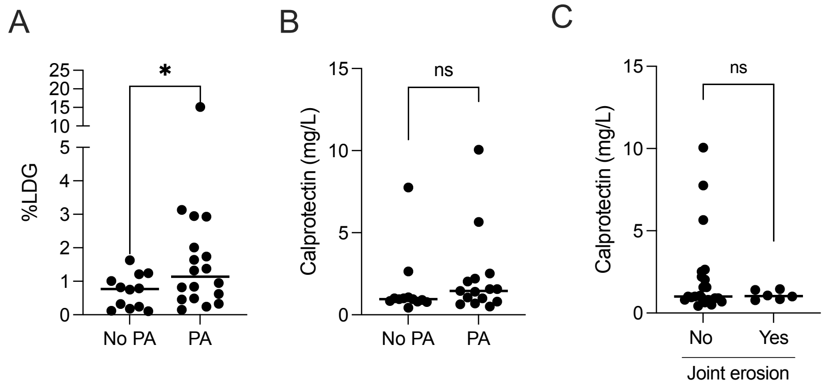

Supplementary Figure S4. Additional univariate analysis of the frequency of low-density neutrophil phenotype (%LDG) **(A)**, levels of the neutrophil-derived proteolytic enzyme calprotectin grouped by presence of PA by HRCT or not **(B)**, and grouped by the presence of joint erosions **(C)**. **P* ≤ 0.05 and ***P* ≤ 0.01 (Mann-Whitney U test)

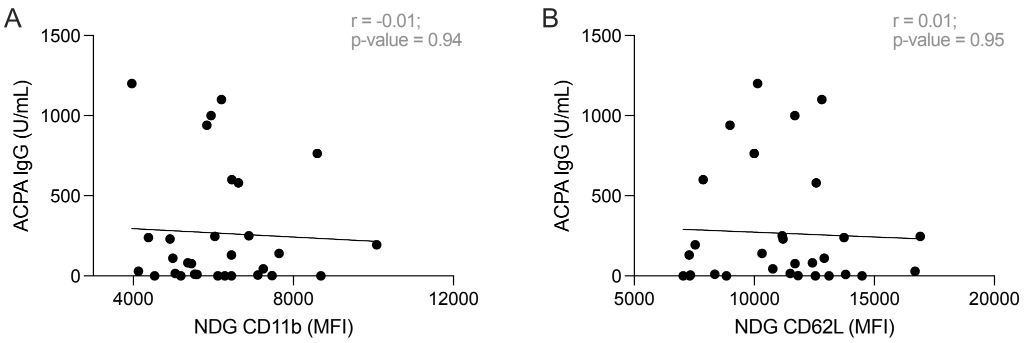

Supplementary Figure S5. Correlation analysis between ACPA IgG levels and neutrophil activation status; NDG CD11b **(A)** and NGD CD62L expression **(B)** in RA patients (n=30) (Spearman's rank correlation test). Linear regression lines are presented in the correlation plots. ACPA = anti-citrullinated protein antibodies. NDG = normal density granulocytes.
