# Supplementary material for: Radiographic airway abnormalities in untreated early rheumatoid arthritis are associated with peripheral neutrophil activation
Source: Arthritis Res Ther. 2023 Mar 20;25:44. doi: 10.1186/s13075-023-03019-5 (PMC10026468; doi:10.1186/s13075-023-03019-5)
Supplement: Supplementary file 2 — Additional file 2: Supplementary table 1. Cluster of differentiation markers used for neutrophil characterization. Supplementary table 2. Demographics and clinical data in patients with versus without RA-Associated PA. [file 13075_2023_3019_MOESM2_ESM.pdf]

Supplementary Table S1. Cluster of differentiation markers used for neutrophil characterization

Marker	Fluorochrome	Clone	Company
CD45	FITC	2D1	Biolegend
CD11b	APC	D12	BD Biosciences
CD62L	PE	SK11	BD Biosciences
CD177	BV421	MEM-166	BD Biosciences
Viability	7AAD		BD Biosciences

Supplementary Table S2. Demographics and clinical data in patients with versus without RA-associated PA

Variables	No pulmonary abnormalities (n = 12)	Pulmonary abnormalities (n = 18)	P-value
Smoking status, n (%)			
Never smoker	7 (58%)	11 (61%)	ns
Former smoker	3 (25%)	3 (17%)	ns
Current smoker	2 (17%)	4 (22%)	ns
CRP, median (range)	5 (0.7-8.3)	11.5 (1-105)	0.0035
ESR, median (range)	16.5 (8-34)	22.5 (2-104)	ns
DAS28, mean (±S.D)	4.6 (±0.90)	5.15 (±1.1)	ns
DAS28-CRP, mean (±S.D)	4.1 (±0.81)	4.92 (±1.0)	0.0417
RF (IgM) titre, mean (±S.D)	8.6 (±8.4)	75.4 (±76.4)	0.0025
ACPA (IgG) titre, mean (±S.D)	162.5 (±312.3)	336.1 (±400.5)	ns
RF and ACPA positive, n (%)[a,b]	3 (25%)	11 (61%)	ns
Calprotectin, median (range)	0.96 (0.43-7.75)	1.46 (0.51-10.5)	ns

CRP = C-reactive protein; DAS28 = disease activity score in 28 joints; RF = Rheumatoid factor; ACPA = anti-citrullinated protein antibodies. Statistical analysis using Mann-Whitney U test for continuous variables and Fisher's exact test for categorial variables.

[a] RF IgM levels >5 kIE/L and/or RF IgA >20 kIE/L are considered positive

[b] ACPA IgG level ≥5 U/mL is considered positive.
